# Aligned Scaffolds with Biomolecular Gradients for Regenerative Medicine

**DOI:** 10.3390/polym11020341

**Published:** 2019-02-15

**Authors:** Xiaoran Li, Zhenni Chen, Haimin Zhang, Yan Zhuang, He Shen, Yanyan Chen, Yannan Zhao, Bing Chen, Zhifeng Xiao, Jianwu Dai

**Affiliations:** 1 Innovation Center for Textile Science and Technology, Donghua University, Shanghai 200051, China; xiaoranli@dhu.edu.cn; 2 CAS Key Laboratory for Nano-Bio Interface Research, Division of Nanobiomedicine, Suzhou Institute of Nano-Tech and Nano-Bionics, Chinese Academy of Sciences, Suzhou 215123, China; Znchen2017@sinano.ac.cn (Z.C.); Hmzhang2017@sinano.ac.cn (H.Z.); Yzhuang2012@sinano.ac.cn (Y.Z.); Hshen2009@sinano.ac.cn (H.S.); Yychen2006@sinano.ac.cn (Y.C.); 3 Nano science and technology institute, University of Science and Technology of China, Suzhou 215123, China; 4 School of Pharmacy, Xi’an Jiaotong University, Xi’an 710061, China; 5 State Key Laboratory of Molecular Developmental Biology, Institute of Genetics and Developmental Biology, Chinese Academy of Sciences, Beijing 100101, China; ynzhao@genetics.ac.cn (Y.Z.); bchen@genetics.ac.cn (B.C.); zfxiao@genetics.ac.cn (Z.X.)

**Keywords:** aligned scaffolds, biomolecular gradients, electrospinning, 3D printing, regenerative medicine

## Abstract

Aligned topography and biomolecular gradients exist in various native tissues and play pivotal roles in a set of biological processes. Scaffolds that recapitulate the complex structure and microenvironment show great potential in promoting tissue regeneration and repair. We begin with a discussion on the fabrication of aligned scaffolds, followed by how biomolecular gradients can be immobilized on aligned scaffolds. In particular, we emphasize how electrospinning, freeze drying, and 3D printing technology can accomplish aligned topography and biomolecular gradients flexibly and robustly. We then highlight several applications of aligned scaffolds and biomolecular gradients in regenerative medicine including nerve, tendon/ligament, and tendon/ligament-to-bone insertion regeneration. Finally, we finish with conclusions and future perspectives on the use of aligned scaffolds with biomolecular gradients in regenerative medicine.

## 1. Introduction

In recent decades, tissue-engineered scaffolds have shown great therapeutic potential in regenerative medicine for improving clinical outcomes. Tissue engineering aims to govern the cellular microenvironment to favor cell assembly and tissue function in order to replace damaged native tissues and guide their regeneration [1]. Many efforts have been made to recapitulate the structure and biology of the native extracellular matrix (ECM) in specific tissues [2].

Living tissues exhibit a specialized architecture and present unique biological cues to ensure specific functions. In the well-organized ECM, both topography and biological signals provide essential guidance cues for tissue orientation and function. Apart from an anisotropic structure, multiple tissues such as nerves, tendons, ligaments, and muscle tissues are composed of perfectly aligned and densely packed fibers. The aligned structure specifies the tissue orientation and function. For example, well aligned cardiomyocytes induce synchronized contraction. More importantly, biological signals play a critical role during tissue development and regeneration. Intriguingly, biological signals distributed in a gradual manner contribute to biological processes, such as embryogenesis and cell migration during tissue regeneration. For example, a linear retinoic acid gradient is present in embryo and is essential for normal embryonic development [3]. As another example, the complementary gradients of sonic hedgehog (Shh) and bone morphogenetic protein (BMP) drive the ventral and dorsal identity of neural progenitors and determine neuronal cell subtypes in a dose-dependent fashion along the gradient at different locations [4]. Moreover, cytokine/chemokine gradients form when tissue injury occurs. For example, after myocardial ischemia injury, formation of gradients of angiogenic factors, such as vascular endothelial growth factor (VEGF), stromal cell derived factor (SDF-1) and monocyte chemoattractant protein-1 (MCP-1), leads to mobilization and recruitment of endothelial progenitor cells from the bone marrow niche to the lesion site for neovasculogenesis [5].

Biomimetic scaffolds capable of imitating aligned structures and biomolecular gradients may be essential for tissue regeneration. Electrospinning provides a simple and versatile method to fabricate uniaxially aligned nanofibers. Structural mimicry of the fibrous network of native ECM and ease of control of fiber organization make electrospun fibrous scaffolds potential candidates for a variety of applications in tissue regeneration. In recent years, advances in 3D printing technology have presented new possibilities for regenerative medicine because this technology enables manufacture of complex structures. In addition, introduction of drug delivery systems into the scaffolds can enhance their therapeutic efficacy. Spatial and temporal control of drug release holds great potential in controlling cell behavior and reconstructing damaged tissues. In addition, stem cell-based therapy offers a promising paradigm for regenerative medicine, and has been advancing rapidly in recent years [6].

The objective of this article is to present recent progress in the fabrication of aligned scaffolds with biomolecular gradients as well as their applications and future directions in regenerative medicine. We first introduced the preparation of aligned scaffolds, and then focus on the fabrication of aligned scaffolds with biomolecular gradients. Next, we highlighted their applications in regenerative medicine including nerve, tendon/ligament, and tendon/ligament-bone insertion regeneration. Finally, the challenges and future directions in the field will be discussed.

## 2. Aligned Scaffolds

Aligned scaffolds hold great potential in regenerative medicine owing to their mimicry of the native structure of specific tissues, ability to control cellular behavior and enhance mechanical properties, and potential to improve biological outcomes. To date, a variety of techniques have been exploited to fabricate aligned scaffolds with different microstructures and architectures.

### 2.1. Electrospinning

Electrospinning has become a versatile method for generating nanoscale fibers [7]. Electrospun nanofibers with high porosity and a large surface area can mimic the fibrous structure of ECM [8]. One of the advantages of electrospinning is easy control over alignment and patterning. Normally, random fibers are obtained on a flat aluminum plate. In contrast, axially aligned fibers can be readily formed on collectors composed of two conductive strips separated by a void gap [9], and a high-speed rotating drum [10]. Rodríguez-Cabello et al. [11] prepared water stable aligned nanofibers by in situ crosslinking of clickable elastin-like recombinamers during the electrospinning flight without the need of crosslinking agents. The aligned nanofibers and coating (such as hyaluronic acid) could promote cell elongation and migration [12]. In addition, radially aligned nanofibers can be readily formed on a collector consisting of a center point electrode and a peripheral ring electrode, and the radially aligned fibers improved migration of dural fibroblasts toward the center [13].

**Figure 1:**
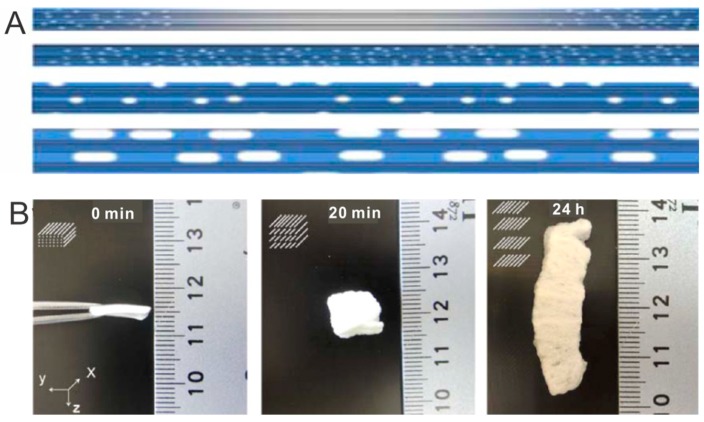
Expanded 3D aligned fibrous scaffolds: (**A**) schematic representation of expansion of 2D nanofiber membranes to 3D fibrous scaffolds using a modified gas-forming technique. (**B**) photographs of aligned PCL fiber mats after treatment with 1 M NaBH_4_ for 0 min, 20 min, and 24 h. Reprinted from Reference [15].

Despite the great progress in the use of nanofibers in regenerative medicine, their application has been extremely limited due to their small pore size and 2D structure. In recent years, breakthroughs in production of 3D fibrous scaffolds have been achieved. For example, Mao et al. [14] created hydrogel microfibers composed of natural polymers with a uniaxial alignment via combination of electrical and mechanical stretching under ambient conditions. Owing to the organic-free processing conditions, live cells were readily incorporated inside the hydrogel fibers in an aligned fashion. As another example, Xie et al. [15,16] reported a modified gas-foaming technique to expand 2D electrospun membranes composed of random or aligned nanofibers into 3D nanofiber scaffolds. In their work, NaBH_4_ was used to generate hydrogen gas bubbles for scaffold expansion (Figure 1A). A significant increase in thickness of the aligned poly (ε-caprolactone) (PCL) nanofiber membrane was achieved from 1 mm to 35.6 mm after 24 h of treatment with 1 M NaBH_4_, leading to successful cell infiltration throughout the bulk of the 3D scaffolds in contrast to the surface cell layer on the 2D membrane (Figure 1B). In their follow-up work [17], expanded aligned nanofiber scaffolds comprising PCL and gelatin were developed for hemostasis. The injectable and superelastic nanofiber scaffolds showed high capacities in blood absorption and whole blood clotting.

Moreover, electrospun nanofibers offer a feasible platform for drug delivery with drugs, peptides or bioactive factors via encapsulation inside the nanofibers or immobilization on the surface of the nanofibers [18]. The ease of control of composition, microstructure, and alignment or patterns enables various drug release profiles, such as burst release, sustained release, biphasic release, and zero-order release [19]. Notably, time-programmed drug release systems have been developed including sequential release and on-demand release [20] by stacking of the nanofibers and incorporation of stimuli-responsive species. The nanofiber functionalization provides a closer resemblance to native ECM or the in vivo microenvironment.

### 2.2. Freeze Drying

**Figure 2:**
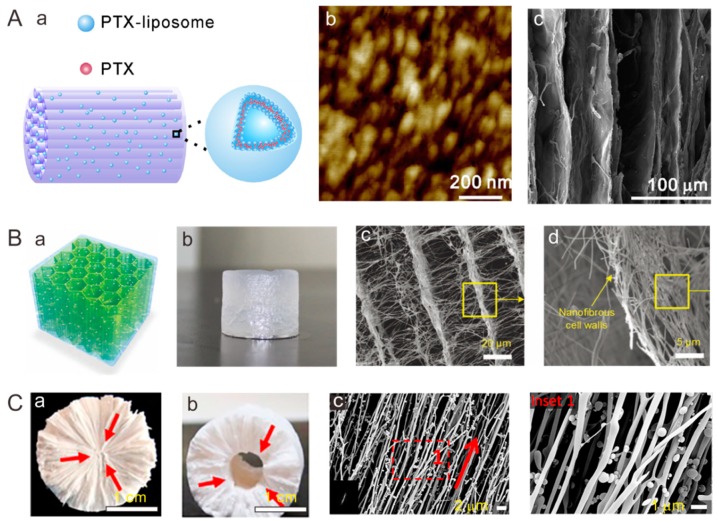
Representative aligned porous scaffolds produced by freeze drying. (**A**) Collagen scaffolds with arrayed microchannels loaded with drug-incorporated liposomes: (**a**) schematic of a drug-liposome loaded oriented collagen scaffold, (**b**) atomic force microscopy image of drug-liposomes, and (**c**) SEM image of an aligned collagen scaffold. Reprinted from Reference [27]. (**B**) 3D aligned cellular scaffold composed of electrospun nanofibers: (**a**) schematic of a nanofibrous scaffold, (**b**) a photograph of a hydrated nanofibrous scaffold, (**c**) SEM image of cellular architecture, and (**d**) SEM image of nanofibrous wall. Reprinted from Reference [29]. (**C**) 3D scaffolds with co-aligned nanofibers and macrochannels: (**a**,**b**) photographs of nanofibrous scaffolds in various geometries (a: cylinder, b: tube), and (**c**) SEM images of aligned silk fibroin nanofibrous scaffolds. Reprinted from Reference [30].

Freeze drying technique offers a feasible tool for fabrication of porous scaffolds owing to rapid dehydration of the wet materials without destruction of their original 3D architecture [21,22]. The formation of cellular structure is based on the water crystallization-induced phase separation. The growth direction and size of the ice crystals can be controlled by the temperature gradient and solution concentration, resulting in uniaxially aligned porous structures [23]. A directional freezing technique has been developed to assemble polymers into 3D porous scaffolds with an aligned lamellar structure [24]. As an extension, a scaffold composed of interconnected radial channels has been fabricated via the freeze-drying method by making use of a round plastic mold with a thin copper rod at the center [25]. With freeze from the center, growing ice crystals expel hydroxyapatite particles, resulting in a lamellar structure parallel to the freezing direction. The obtained radially aligned scaffolds promoted self-seeding of cells attributed to the gradient channel structure-induced spontaneous capillary effect. Dai et al. [26] produced sponge-like porous collagen scaffolds consisting of packed aligned tubular pores with a diameter of 10–30 µm. In their follow-up work [27], a functional collagen scaffold was developed by encapsulating drug-incorporated liposomes into the collagen scaffolds with arrayed microchannels to alleviate the adverse microenvironment of the lesion site (Figure 2A). As another strategy, Ding et al. [28] developed an electrospun nanofiber-assembled 3D cellular scaffold via freeze drying of short electrospun nanofibers dispersions obtained by high speed homogenization, followed by crosslinking to achieve physical–physical bonding among the fibers. Notably, scaffolds with an aligned cellular architecture can be formed with the nanofibrous network on the cell wall (Figure 2B) [29]. Three-dimensional fibrous scaffolds consisting of alginate and SiO_2_ nanofibers showed great super-elastic and shape memory capabilities. Owing to the open cellular structure and interconnected pores, freeze-dried scaffolds are well-suited for cell delivery. In addition, 3D scaffolds composed of both aligned nanofibers and aligned macrochannels were fabricated via manipulation of ice crystallization (Figure 2C) [30]. The 3D scaffolds could be formed in various geometries (Figure 2Ca,b), and the nanofibers were well aligned along the radial direction of cylinder scaffolds (Figure 2Cc). One future direction would be to achieve a high degree of control over the porous and lamellar structure.

### 2.3. 3D Printing

**Figure 3:**
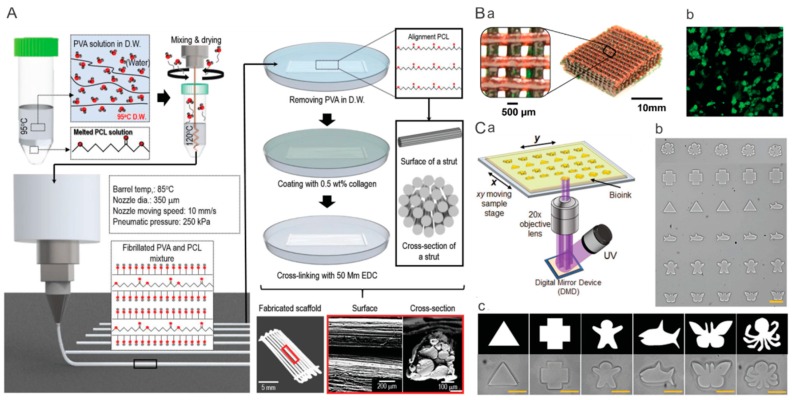
Representative 3D complex scaffolds prepared by 3D printing. (**A**) Schematic of fabrication process and photograph/SEM images of aligned microfibrillated PCL/collagen scaffold. Reprinted from Reference [34]. (**B**) Extrusion-based 3D printing: (**a**) 3D printed hydrogel, (**b**) live–dead assay, and (**c**) viability of encapsulated human embryonic kidney cells. Reprinted from Reference [35]. (**C**) Digital light processing-based 3D printing: (**a**) schematic of digital light processing system, and fabricated microgels (**b**) with complex shapes and (**c**) in numerous arrays. Reprinted from Reference [39].

The emerging 3D printing technique has become a precise and personalized approach for producing customizable tissues or organs that can recapitulate the intricate native construct and biological activity [31,32]. Currently, the most popular strategy is direct 3D printing, which can print bioinks into complex architectures composed of microfibers [33]. For instance, Kim et al. fabricated an aligned microfibrous PCL bundle via a combinatory process of microfibrillation of PVA/PCL mixture and leaching process of poly(vinyl alcohol) (PVA) from PVA/PCL microfibers to imitate microfibril muscle structure. In addition, type-I collagen was supplemented on the PCL microfibers to improve the cellular activities (Figure 3A) [34]. As another example, stretchable hydrogels of covalently crosslinked PEG and ionically crosslinked alginate was prepared through UV irradiation and Ca^2+^ exposure, respectively [35]. The 3D printed hydrogel was composed of a patterned filament network (Figure 3Ba), and a high viability of encapsulated human embryonic kidney cells was found over 7 d of culture (Figure 3Bb,c). Another promising strategy is fabrication of complex constructs by making use of 3D printed geometric sacrifices. Based on this strategy, Kelly et al. [36] developed a gelatin-methacryloyl (GelMA) hydrogel scaffold with interconnected microchannel networks inside to induce vascularization during bone repair. In brief, a pluronic sacrifice was printed into a free-standing network of micropillars, followed by casting of mesenchymal stem cell (MSC)-laden GelMA solution into the pluronic model and crosslinking of GelMA via UV exposure. After sacrifice of pluronic micropillars in medium, a hydrogel with interconnected microchannels was formed. When the scaffolds were implanted into critically sized femoral bone defects in rats, constructs with microchannels facilitated osteoclast/immune cell invasion, hydrogel degradation and vascularization.

Modification with bioactive molecules and encapsulation of cells or stem cells into hydrogel scaffolds significantly stimulates the tissue regeneration [37]. The extrusion-based 3D printing has intrinsic limitations of large mechanical stress and long printing time which would reduce viability of encapsulated cells. The development of digital light processing (DLP) technique enables creating complex 3D constructs via photopolymerization under UV exposure in a layer-by-layer fashion with high speed and high resolution (~1 µm) [38]. Additionally, owing to short-time printing and nozzle-free printing technique, DLP possesses the capability of increasing cell viability. As shown in Figure 3C, by making use of a digital micromirror device (DMD) to reflect the micropatterned UV light on to the bioink, microgels with complex patterns can be obtained [39]. Based on DLP strategy, Lee et al. [40] produced highly complex organ structures, including heart, vessel, brain, trachea, and ear with biocompatibility.

Despite the recent success in printing 3D constructs, toxic agents or harsh conditions severely restrain their ability to encapsulate cells. Additionally, the lack of sufficient mechanical strength and print resolution has limited their application. Therefore, one interesting future possibility is to explore printable bioinks or in situ crosslinking reaction of the bioinks to improve the biocompatibility, resolution, and mechanical properties of the 3D printed scaffolds.

## 3. Aligned Scaffolds with Biomolecular Gradients

In addition to an aligned topography, a gradient of bioactive signals has a profound effect on cell stretching, migration, and differentiation of stem cells and on tissue regeneration. Therefore, many efforts have been devoted to developing aligned scaffolds with biomolecular gradients.

### 3.1. Direct Electrospinning

**Figure 4:**
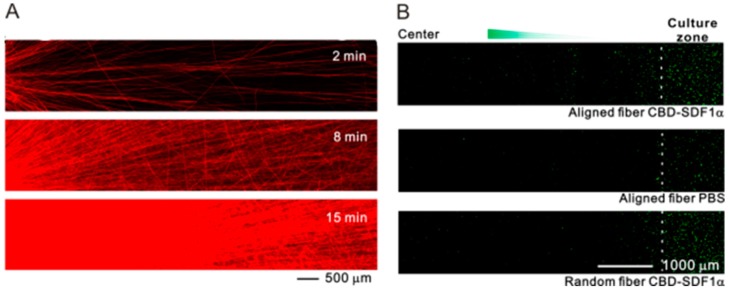
Radially aligned nanofibers with an immobilized SDF 1α gradient produced by electrospinning. (**A**) Gradient patterns can be controlled by adjusting the collection time during electrospinning. (**B**) SDF 1α gradient and aligned fibers improved NSC migration synergistically. Reprinted from Reference [43].

Despite the success in aligned electrospun nanofibers, development of biomolecular gradients along the aligned nanofibers still faces great challenges. A dual-gradient of fiber density and bioactive molecules has been generated by Dinis et al. [41]. In this study, aligned protein-encapsulated silk electrospun fibers were prepared on a coverslip, and the fibers located on the first 4 mm of the coverslip were retained, while the rest of the fibers were discarded. The process was repeated with lower protein concentration and a larger retaining region in four successive electrospinning processes. The obtained nerve growth factor (NGF) gradient nanofibers induced oriented and enhanced growth of DRG neurons. In another study, a combined strategy of in-line electrospinning and air-gap electrospinning was demonstrated to be feasible in fabrication of biomolecular gradients along aligned electrospun nanofibers [42]. However, these methods are not suitable for fabrication of continuous gradients along aligned electrospun nanofibers. In addition, the process is tedious and time-consuming. To address this limitation, Dai et al. [43] demonstrated a facile method for fabrication of continuous biomolecular gradients embedded in radially aligned electrospun nanofibers. Attributed to the gradual decrease in fiber density from the center to the periphery of the radially aligned nanofiber membrane, a continuous biomolecular gradient along the aligned fibers readily formed (Figure 4A), and the gradient patterns could be controlled by adjusting collection time, and collector size during electrospinning. The stromal cell derived factor-1α (SDF 1α) gradients immobilized on the fibers directed and accelerated the migration of neural stem cells (NSCs) from the periphery to the center along the aligner fibers (Figure 4B). This kind of SDF 1α gradient scaffolds could recruit endogenous NSCs and benefit in situ spinal cord injury repair.

### 3.2. Direct 3D Printing

**Figure 5:**
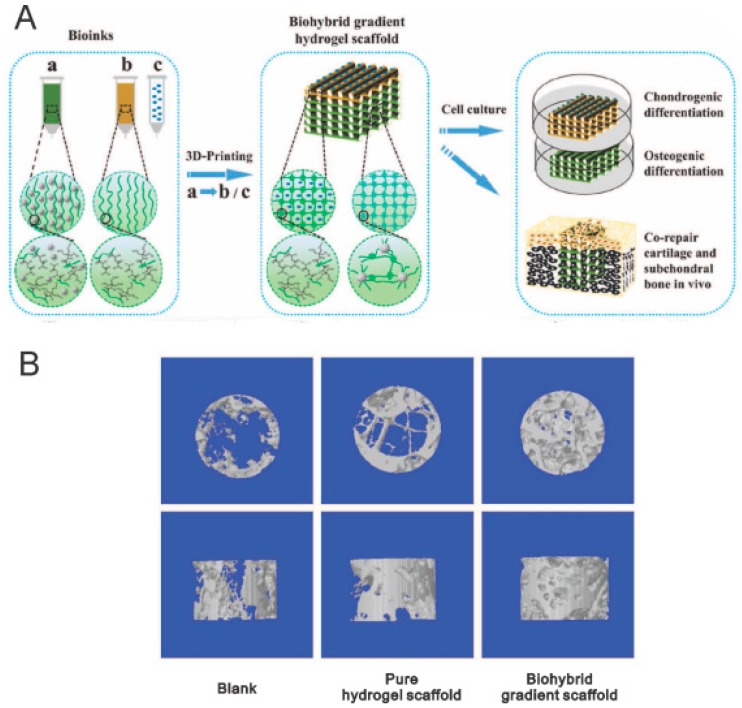
Bilayer hydrogel scaffolds with compositional gradients prepared by 3D printing for repair of osteochondral defect. (**A**) Schematic illustration of 3D printed biohybrid gradient scaffolds. (**B**) 3D printed gradient scaffolds stimulated cartilage and subchondral bone regeneration simultaneously in a rat osteochondral defect model. Reprinted from Reference [45].

The 3D printing technique opens up a new avenue for producing gradual scaffolds, and enable incorporation of physical, chemical, biological, and cellular gradients into scaffolds to recapitulate the heterogeneous environment of native tissues/organs [44]. To mimic articular cartilage-subchondral bone architecture, Liu et al. [45] prepared bilayer hydrogel scaffolds with gradients in composition comprising transforming growth factor (TGF)-β1 loaded into the top layers and β-tricalciumphosphate (β-TCP) incorporated into the bottom layers (Figure 5A). It was found that the TGF-β1 layer enhanced the growth and chondrogenic differentiation of human bone marrow stem cells (hBMSCs), whereas the β-TCP layer promoted proliferation and osteogenic differentiation of hBMSCs. The in vivo study verified that the 3D printed gradient scaffolds facilitated simultaneous regeneration of cartilage and subchondral bone in a rat osteochondral defect model (Figure 5B). Although the proof of concept of 3D printing has been widely accepted and successfully demonstrated in vitro and in vivo, there is still a long way to go before wide use of 3D printing in clinical practice.

### 3.3. Post-Processing Treatment on Aligned Scaffolds–Inkjet Printing

**Figure 6:**
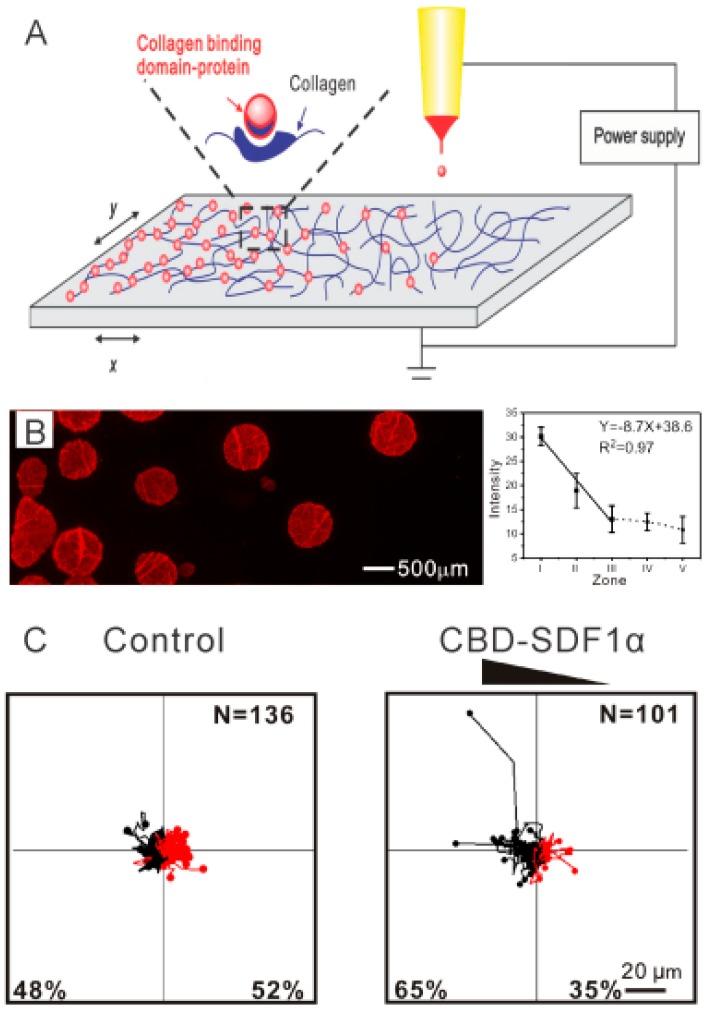
CBD-SDF1α gradients produced by inkjet printing on electrospun nanofibers. (**A**) Schematic illustration of inkjet printing. (**B**) CBD-SDF1α gradient patterns on collagen nanofibers. (**C**) A CBD-SDF1α gradient promoted NSC migration. Reprinted from Reference [51].

The inkjet printing technique provides a sophisticated and versatile method for produce spatial coating on a scaffold surface [46]. A biomolecular solution [47] or living cells [48] can be used as bioink for bioprinting, the droplet pattern is programed and the deposition concentration is modulated by the original concentration and overprinting. For instance, to demonstrate that cell alignment and cell differentiation can be controlled simultaneously by aligned fibers and spatial patterning of growth factors, spatially patterned fibroblast growth factor-2 (FGF-2) and BMP-2 were printed on oriented fibers using an inkjet-based bioprinter [49]. It was found that FGF-2 and BMP-2 patterns stimulated tenocyte and osteoblast fates of stem cells, respectively, along with cell alignment. Similarly, patterning of biomolecular droplets with different shapes can be produced on electrospun nanofibers with a high printing accuracy [50]. In an endeavor to explore biomolecular gradients, Dai et al. [51] developed a gradual patterning of biomolecules on collagen electrospun nanofibers by adjusting the translation speed of substrate in X and Y directions in a controllable fashion (Figure 6A,B). To prolong the retention time of the gradient, SDF1α was fused with a unique peptide of collagen-binding domain (CBD), which is specifically able to bind to collagen. The CBD-SDF1α gradients with various patterns induced NSC migration toward the region with a higher CBD-SDF1α content (Figure 6C). Surface deposition poses a challenge for long-term gradual release and viability in vivo. Thus, in the future, hydrogel or particles can be introduced as vehicles for the biomolecules.

### 3.4. Post-Processing Treatment on Aligned Scaffolds–Gradual Infusion

**Figure 7:**
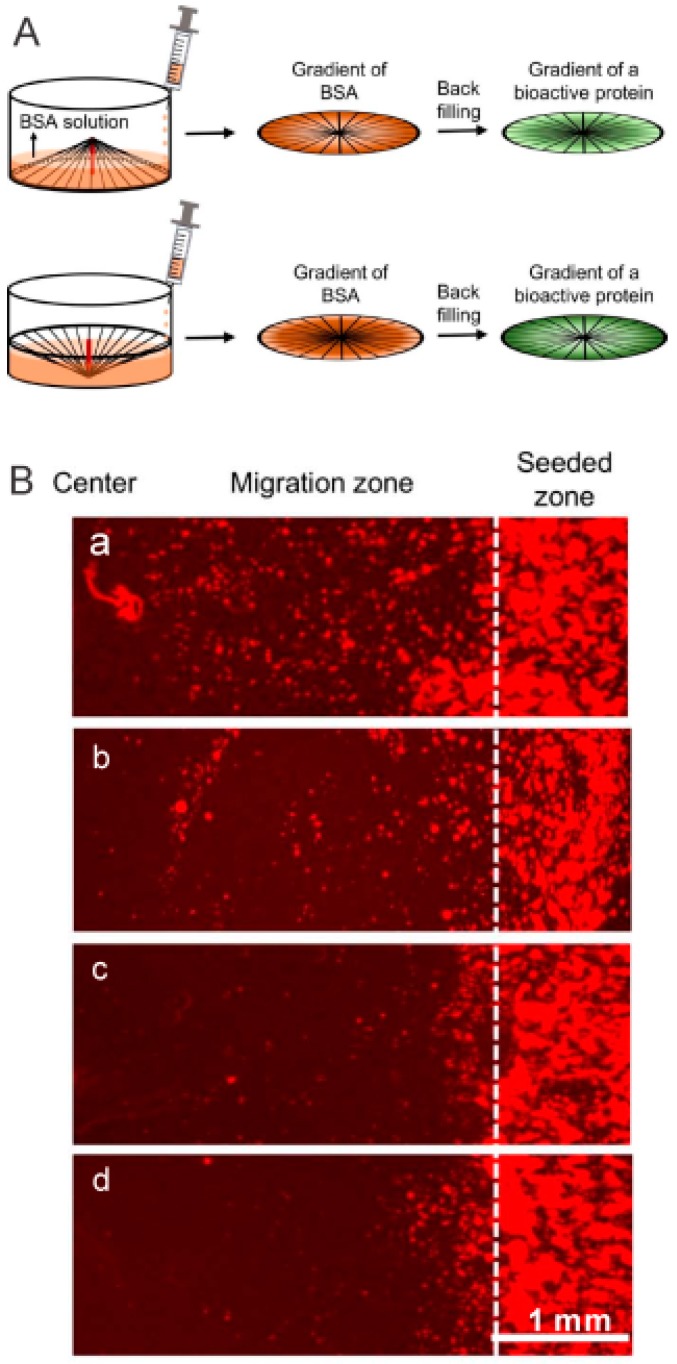
Circular gradients in electrospun nanofibers generated by the infusion method. (**A**) Schematic illustration of the infusion method to fabricate circular gradients. (**B**) EGF gradients on radially aligned nanofibers promoted migration of keratinocytes after 3 d of culture: (**a**) a graded coating of EGF, (**b**) a uniform coating of EGF, (**c**) a graded coating of BSA, and (**d**) bare scaffolds. Reprinted from References [55].

Gradual infusion offers a straightforward approach to produce gradients in scaffolds. For example, Xia et al. [52] constructed electrospun nanofibrous scaffolds with a continuous mineral content gradation by infusion of simulated body fluid (SBF) to mimic tendon to bone insertion sites. As an extension, electrospun nanofibrous scaffolds with a continuous gradation in mineral content and biological cues have been developed by Li et al. [53]. In their study, gradients in amino groups of poly (lactide-*co*-glycolide) (PLGA) electrospun nanofibers were obtained through an aminolysis process by the infusion method. Subsequently, gradients in gelatin density, hydroxyapatite amount, and plasmid (pDNA) loading contents were achieved. Based on the infusion strategy, Gao et al. [54] produced complementary density gradients for selective and directional migration of cells. In a follow-up study by Xia et al. [55], they generated circular protein gradients on radially aligned fibrous scaffolds (Figure 7A). The gradients in laminin and epidermal growth factor (EGF) promoted migration of fibroblasts and keratinocytes respectively (Figure 7B), and an NGF gradient improved neurite extension. The infusion method is simple and robust, allowing scale-up manufacturing. However, it poses difficulties in preciseness and reproductivity.

## 4. Applications in Regenerative Medicine

### 4.1. Nerve

**Figure 8:**
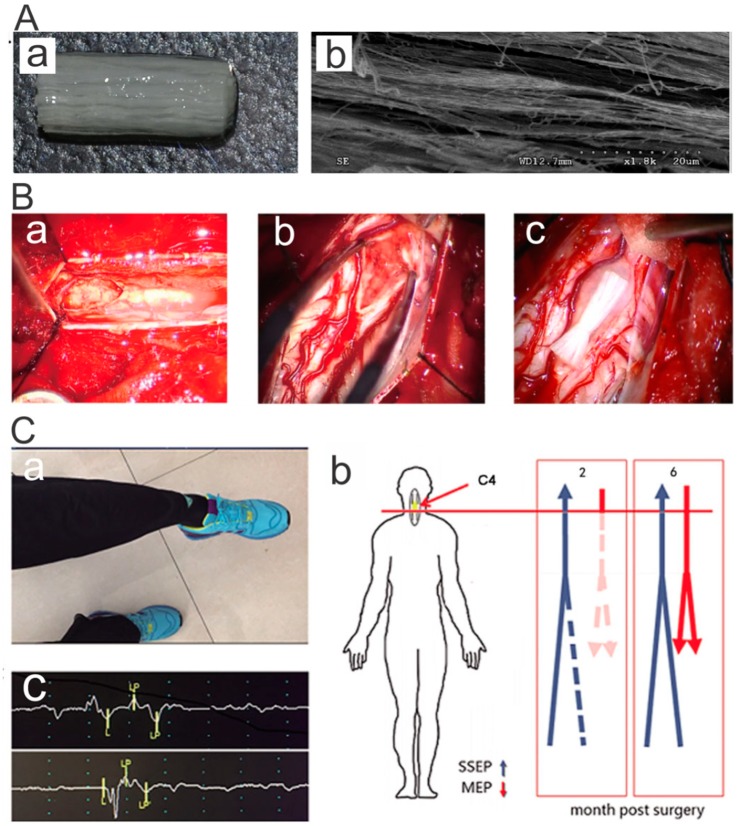
Aligned collagen microfibers promoted functional recovery of SCI patients. (**A**) (**a**) Photograph and (**b**) SEM image of a NeuroRegen scaffold composed of aligned microfibers. Reprinted from Reference [63]. (**B**) Intraoperative photographs of the SCI site. The lesion site was filled with necrosis tissue in (**a**) a thoracic SCI patient, and (**b**) a cervical SCI patient. (**c**) NeuroRegen scaffold transplantation into the spinal cord gap of a cervical SCI patient. Reprinted from Reference [71]. (**C**) NeuroRegen scaffolds with MSC transplantation improved the functional recovery of acute cervical SCI patients. Reprinted from Reference [71].

Spinal cord injury (SCI) often results in serious impairment of motor and sensory functions. The spinal cord consists of millions of nerve fibers grouped in bundles that transmit electrical impulse to the tissues [56]. Aligned fibers that can mimic nerve fibers would provide directional cues to promote outgrowth and extension of axons. It is becoming increasingly clear that an inhibitory microenvironment composed of scar tissue and myelin proteins hinders nerve regeneration and neuronal differentiation of neural stem cells [57]. Scaffolds functionalized with biomolecules and stem cells would provide a permissive microenvironment and facilitate SCI recovery [58,59]. Dai et al. have developed various types of aligned collagen scaffolds for SCI repair, including a collagen sponge with arrayed channels [60] and a linearly ordered collagen fibrous scaffold [61] termed as a NeuroRegen scaffold (Figure 8A) [62,63], that promoted axon outgrowth, reduced scar formation, and improved SCI repair. In the past ten years, the NeuroRegen scaffold has been functionalized with biomolecules including neurotropin factors, antibodies, and stem cells including NSCs [64] and MSCs [65,66], and it was found that axon outgrowth, neural differentiation of transplanted [64] and endogenous stem cells [67,68], electrophysiological and motor functional recovery could be achieved in both rat [63,64] and canine [65,66,69,70] SCI models. Importantly, they conducted one-year clinical studies of NeuroRegen scaffold loaded with autologous bone marrow mononuclear cells (BMMCs) [62] or human umbilical cord mesenchymal stem cells (hUCB-MSCs) [56] for repair of chronic complete SCI. At 1 year post-surgery, no adverse effects was found, and partial recovery of sensation and motor function was observed. In their follow-up study, the acute complete SCI patients were implanted with hUCB-MSCs loaded NeuroRegen scaffolds [71]. At 1 year post-surgery, in addition to a significant improvement in sensory and motor functions, the injury status of the two patients was improved from American Spinal Injury Association (ASIA) A complete injury to ASIA C incomplete injury (Figure 8C). Thus, this kind of linearly ordered collagen scaffold holds great promise for clinical use in SCI patients.

**Figure 9:**
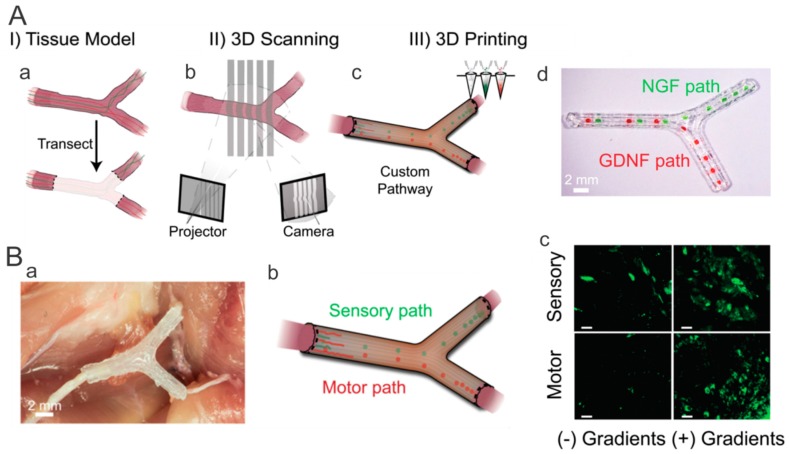
3D printed hollow nerve pathway with spatial growth factors encouraged nerve regeneration. (**A**) Personalized nerve regeneration pathways produced by 3D scanning and printing: (**a**) the tissue model for subsequent imaging, (**b**) image of intact or transected tissue obtained by using structured light scanning, (**c**) the 3D printed nerve pathway, and (**d**) representative photograph of the 3D printed pathway with gradient patterns of NGF and GDNF. (**B**) In vivo study showing sensory and motor regeneration with the 3D printed nerve pathways: (**a**) photograph of an implanted 3D printed nerve pathway, (**b**) schematic of hollow gradient nerve pathway for bifurcation into sensory and motor nerve paths, and (**c**) histology of regenerated sensory and motor nerves stained for tubulin (green). Reprinted from Reference [72].

Three-dimensional printing opens up new avenues for fabrication of complex nerve repair scaffolds. McAlpine and Jia et al. [72] produced custom nerve scaffolds with aligned microgrooves as the physical cues, and spatially controlled multicomponent gradients as the biomolecular cues. Specifically, NGF and glial cell line-derived neurotrophic factor (GDNF) were selected as the sensory and motor path cues respectively (Figure 9A). The in vitro studies showed that the aligned physical cues and gradual biomolecular cues facilitated axonal outgrowth and chemoattractant /chemokinetic functionality, and the 3D printed scaffolds stimulated regeneration of complex nerves after transplantation in a 10-mm complex nerve gap in rats (Figure 9B).

In recent years, NSCs and MSCs have been widely employed in nerve repair. Despite the progress in nerve scaffolds, the detailed mechanisms underlying nerve regeneration, recruitment of endogenous stem cells, and the in vivo fate of transplanted stem cells remain poorly understood. Future work on the underlying mechanisms would provide a theoretical basis for scaffold fabrication.

### 4.2. Tendon

Tendon, composed of tendon cells distributed in an aligned pattern of type I collagen fibrils, is essential for transmitting stress. However, it has poor intrinsic healing capacity due to their limited vascularity. In addition, the scar-like tissue formed in the natural healing possesses inferior mechanical properties. The tissue engineering strategy enables rapid tendon healing with adequate mechanical properties. Aligned fibers can closely mimic the structural anisotropy and mechanical properties. Interestingly, crimped nanofibers have been generated by controlling the degree of shrinkage via ethanol treatment, and the crimped electrospun nanofibers showed the mechanical characteristics of native tendon tissues [73]. Aligned multilayered electrospun nanofibrous scaffolds have been demonstrated to improve collagen alignment, tendon-related gene expression, and mechanical properties [74]. In addition, freeze-dried scaffolds with longitudinally aligned tracks promoted attachment, metabolic activity and alignment of equine tendon cells [75]. In addition, supplementation of platelet-derived growth factor-BB (PDGF-BB) and insulin-like growth factor 1 (IGF-1) was found to improve tendon cell motility, viability, and tendon gene expression.

Stem cell potential in tendon tissue engineering has also been investigated [76]. The effect of collagenous matrices derived from tendon, bone, and dermis tissues on differentiation of human tendon stem/progenitor cells (hTSPCs) and tendon repair were investigated [77]. Surprisingly, a tendon-derived decellularized matrix encouraged tenogenic-lineage differentiation of hTSPCs, rather than osteogenesis even under osteogenic induction conditions. In contrast, bone-derived decellularized matrix promoted osteogenic differentiation of hTSPCs, whereas dermal skin-derived collagen matrix had no apparent effect on hTSPC differentiation. Tendon-derived decellularized matrix with hTSPCs synergistically accelerated rat Achilles tendon injury repair. Ouyang et al. [78] reported that well-aligned electrospun nanofibers induced the differentiation of human-induced pluripotent stem cells (hiPSC)-induced MSCs into tenocyte-like cells through mechanical-signal pathway. A rat Achilles tendon injury repair model confirmed that the aligned fibrous scaffold with hiPSC-MSCs had better mechanical properties, and promoted tendon repair. Researchers may consider further improving the mechanical properties of the repaired tendon/ligament in the future study.

### 4.3. Tendon/Ligament to Bone Insertion Site

The native tendon/ligament to bone interface exhibits gradients in collagen fiber orientation, mineral content and cell type [79,80]. Repair of tendon/ligament to bone insertion site is a grand challenge due to the stress of the soft-hard tissue interface. Various approaches have been attempted to fabricate gradient scaffolds for improving engraftment of the tendon/ligament to bone [81]. For mimicking the structural gradient of collagen fibers at the tendon to bone insertion site, a “random-aligned-random” ECM scaffold was produced via treatment with acetic acid and ultrasound on the two ends of the ECM scaffolds [82]. After transplantation in a rabbit anterior cruciate ligament (ACL) reconstruction model, it was found that the “random-aligned-random” ECM scaffold stimulated bone and fibrocartilage formation at the interface site compared with the unmodified tendon ECM scaffolds. Gradient in both mineral content and cell phenotypes on aligned electrospun nanofibers was developed, in which a graded mineral coating on the nanofibers induced osteogenic differentiation of adipose-derived mesenchymal stem cells (ASCs) spatially [83]. As a step forward in engineering the insertion site, electrospun nanofibrous scaffolds with structure, composition and biological gradients were developed to fabricate a graft mimicking bone–tendon–bone insertion site [84]. In this study, random-aligned-random oriented nanofibers were fabricated firstly to mimic the topography structure. Then PDGF-BB was immobilized with higher content at the center aligned part to induce tenogenic differentiation of ASCs in a gradient fashion. And finally mineralization at both ends of the nanofibers was achieved by controlling immersion time in SBF with an infusion method. Alizarin staining was restricted at the mineralized random part, and SEM image confirmed the mineral deposition at the two ends of the scaffolds with random nanofibers. Despite these big advances, these methods are inadequate to comprehensively and precisely mimic the complex structure, component and biological cues of the insertion site. In future work, customizing the gradient composite scaffolds for specific locations would be beneficial.

## 5. Conclusions and Future Perspective

Advances in manufacturing techniques such as electrospinning, freeze drying, and 3D printing enable the development of aligned scaffolds with biomolecular gradients that can better recapitulate the architecture and microenvironment of native tissues/organs. The aligned topography with oriented patterns in native tissues contributes to cell migration, mechanical loading, and signal transfer. A gradual transition in cell type and composition exists in interfacial tissues [85]. Spatial patterning of biological cues plays a crucial role in embryogenesis and tissue self-regeneration. Inspired by the native system, biomimetic scaffolds with varying structures, compositions, and biological cues have emerged as a potential class of biomaterials for regenerative medicine.

Aligned electrospun fiber-based scaffolds offer better mimicry of the structure of oriented native ECM. Especially, the development of electrospun nanofiber assembled 3D scaffolds via a combinational method of electrospinning and freeze drying will greatly expand their biomedical applications. The introduction of spatially distributed biomolecules into the aligned scaffolds stimulates robust tissue regeneration. Unsurprisingly, biomimetic scaffolds present a superior capacity to promote nerve, tendon/ligament and tendon/ligament to bone insertion site regeneration. However, full functional recovery remains a therapeutic challenge. The development of 3D printing technique brings great hope for fabrication of biomimetic scaffolds in a personalized, customized, and accurate approach [86,87,88]. Biomimetic physical cues, such as aligned topography, and specific biological cues, such as biomolecular gradients, can be incorporated into the scaffolds simultaneously via multicomponent printing [89], which offers considerable opportunities for personalized regenerative medicine. Although 3D printing technique has revolutionized the manufacturing process, their applications in regenerative medicine have been restricted by the bioink, i.e., printable biomaterials. A compromise must be reached between printability, curing rate of the fluid, and the mechanical properties of the solid. Future work may explore printable biomaterials and crosslinking methods. Beyond 3D printing, 4D printing has emerged in which the configuration of 3D printed constructs transforms over time upon environmental stimulation [90]. The 4D printed scaffolds hold great potential for use in tissue engineering, biomedical devices, and drug delivery.

Given the complexity of the tissue regeneration process, multiple drugs are needed at different periods. In the future, stimuli-responsive polymers can be introduced into the scaffolds, leading to on-demand drug release. Another future interesting direction would combine different delivery systems, such as nanofibers, spheres, liposomes, and hydrogels with biomimetic scaffolds, resulting in programmed drug release. In recent years, stem cell therapy has become a promising approach in regenerative medicine owing to their ability to improve the microenvironment and direct differentiation of stem cells at the lesion site. However, the current stem cell approach for tissue repair has shown limited success, a better understanding of the mechanism of stem-cell-induced tissue regeneration will benefit the design of biomimetic scaffolds for regenerative medicine. Optimized biomimetic scaffolds hold promise for future therapeutic achievements, with the aim of achieving satisfactory clinical outcomes.
